# Effects of Antiepileptic Drug Monotherapy on One-Carbon Metabolism and DNA Methylation in Patients with Epilepsy

**DOI:** 10.1371/journal.pone.0125656

**Published:** 2015-04-27

**Authors:** Guanzhong Ni, Jiaming Qin, Hongliang Li, Ziyi Chen, Yafang Zhou, Ziyan Fang, Yishu Chen, Jueqian Zhou, Min Huang, Liemin Zhou

**Affiliations:** 1 Department of Neurology, The First Affiliated Hospital, Sun Yat-Sen University, Guangzhou, Guangdong Province, China; 2 Institute of Clinical Pharmacology, School of Pharmaceutical Sciences, Sun Yat-Sen University, Guangzhou, Guangdong Province, China; Queen's University Belfast, UNITED KINGDOM

## Abstract

**Purpose:**

The aim of this study was to compare the serum levels of one-carbon metabolism (OCM) nutrients (e.g., folate, homocysteine and vitamin B12) and peripheral blood DNA methylation in epileptic patients under treatment with antiepileptic drugs (AEDs) and in healthy controls.

**Methods:**

In this cross-sectional study, 60 patients with epilepsy who were receiving valproate (VPA) (n = 30) or lamotrigine (LTG) (n = 30) monotherapy were enrolled. Thirty age and sex matched healthy subjects served as the controls. Serum concentrations of OCM nutrients and peripheral blood DNA methylation status were measured.

**Results:**

Compared to the control group, the VPA group had higher serum levels of homocysteine (p<0.05). No difference in homocysteine concentration was observed in the LTG group. Patients receiving VPA or LTG had significantly lower serum folate levels in comparison with controls (p<0.001). The level of methylation of long interspersed nucleotide element-1 (LINE-1) in peripheral blood was not significantly different between the AED monotherapy group and healthy controls. A difference in the methylation levels of methylenetetrahydrofolate reductase (MTHFR) amplicon was observed between AED-treated patients with epilepsy and controls (p<0.01). A positive correlation between serum folate levels and peripheral blood MTHFR amplicon methylation status was also observed (r = 0.25, p = 0.023).

**Conclusion:**

Our findings suggest that the effects of AED monotherapy on OCM may induce specific regions of DNA hypomethylation.

## Introduction

Long-term or lifelong antiepileptic drug (AED) treatment is usually indispensable for most patients with epilepsy. However, long-time AED therapy is often associated with a wide range of chronic adverse effects, including metabolic and endocrine disturbances, behavioral or psychiatric problems, negative cognitive effects and major congenital malformations [1–4]. Folate (FA), a mediator for the transfer of one-carbon units, is involved in DNA synthesis and methylation. Homocysteine (Hcy), a thiol-containing amino acid formed by demethylation of methionine, is an intermediate product in one-carbon metabolism(OCM). Various studies have shown that treatment with both older and new AEDs may interfere with Hcy metabolism by affecting the levels of FA and vitamin B12 in patients with epilepsy [5, 6]. The literature holds controversial views on OCM in patients with epilepsy under treatment with valproate (VPA). Various results of FA and Hcy concentrations have been reported in VPA-treated patients with epilepsy [7–9]. This discrepancy may be due to differences in age, diet, ethnic origin and duration of medication.

DNA methylation, one of several post-replication epigenetic changes that occurs in the genome, contributes to the regulation of gene expression and maintenance of genome integrity and stability [10]. DNA methylation can be modified by nutrients involved in OCM (e.g., FA, Hcy and vitamin B12) and disturbances in methylation reactions can be caused by the abnormal status of these nutrients [11]. The methylation level of long interspersed nucleotide element-1 (LINE-1) can serve as a surrogate marker of global genomic DNA methylation [12]. Chang et al. found that LINE-1 methylation level was positively correlated with FA content, and negatively correlated with Hcy content [13]. Methylenetetrahydrofolate reductase (MTHFR), which catalyzes the irreversible conversion of 5,10-methylenetetrahydrofolate to 5-methyltetrahydrofolate, is considered a key enzyme in OCM.

In a previous study, prenatal AED exposure was associated with differences in the methylation patterns of cord blood DNA, while most of the subjects who were taking AEDs were exposed to lamotrigine (LTG) [14]. Moreover, another study suggested that VPA could induce a decrease in global methylation in lymphomonocytes DNA of patients with epilepsy [15]. We hypothesized that AEDs affect gene-specific DNA methylation in patients with epilepsy by affecting OCM. Therefore, the aim of the present study was to evaluate the nutrients involved in OCM (e.g., FA, Hcy and vitamin B12), the methylation levels of LINE-1 and MTHFR amplicon in epileptic patients receiving VPA or LTG monotherapy, and to investigate the relationship between the levels of OCM nutrients and DNA methylation.

## Patients and Methods

### Patients

A total of 60 patients with epilepsy (aged between 16 and 55 years) who were treated with VPA or LTG as monotherapy for at least six months were included in this study. The VPA and LTG groups consisted of 30 patients each. Patients who had discontinued their medication or who were treated with other AEDs were excluded from the study. Cases of epilepsy that were caused by ischemic stroke, history of cardiac and peripheral vascular disease, diabetes mellitus, tobacco use, hematologic diseases, endocrine disorders, tumors, pregnancy, liver or renal diseases were excluded from the study. A total of 30 healthy volunteers who received annual physical checkups were recruited to serve as controls. To exclude the potential effects of differences in dietary habits and FA fortification, both of patients and controls were from the same geographic area and were matched for age, sex and ethnic background. All subjects using FA antagonists and vitamins, as well as vegetarians were excluded. The current study was approved by the human ethics committee of the first affiliated hospital, Sun Yat-Sen University, and written informed consent was obtained from each participant.

### Laboratory tests and serum concentration assay

Blood samples were collected from subjects for laboratory evaluations between 08:00 and 08:30 AM after overnight fasting. The levels of serum FA, Hcy and vitamin B12 were measured using the autoanalyser Immulite 2000, by using suitable kits (DPC Diagnostic Products Corporation, Los Angeles, USA) and according to its user guide kit using the immune-assay method.

The serum concentrations of VPA and LTG were assayed using a high-performance liquid chromatographic technique with an ultraviolet detector (Chromsystems, Waters Company, Milford Massachusetts, USA).

### DNA extraction and sodium bisulfite conversion

Whole-blood genomic DNA was extracted from the blood samples using a QIA amp DNA Mini Kit according to the manufacturer’s instructions (Qiagen, Hilden, Germany). The concentration and purity of the DNA were determined by absorbance at 260 and 280 nm using a NanoDrop 1000 Spectrophotometer (Thermo Scientific, Wilmington, USA). Extracted whole-blood genomic DNA was treated with sodium bisulfite using the EZ 96 DNA-methylation kit (Zymo Research, Irvine, CA, USA). The bisulfite converted DNA was re-suspended in 10μl elution buffer and stored at −80°C until analysis.

### MassARRAY quantitative methylation analysis

The Sequenom MassARRAY platform was used to perform the quantitative methylation analysis of the LINE-1 elements and the MTHFR amplicon. The bisulfite converted DNA was followed by PCR amplification (the PCR primers used in the system were designed using Epidesigner online and are shown in **Table 1**), fragmentation after transcription and analysis using a mass spectrometer (Sequenom, Inc, San Diego, USA). This technique generated mass signal patterns that were translated into quantitative DNA-methylation levels of different CpG sites of the LINE-1 element and the MTHFR amplicon by MassARRAY EpiTYPER Analyzer software (version 1.0, Sequenom, Inc, San Diego, USA). Measurements were conducted in triplicate for DNA from the same bisulfite treatment batch on different PCR plates. The methylation level was expressed as the percentage of methylated cytosines over the total number of methylated and unmethylated cytosines. Prior to analysis, strict quality control was carried out to remove potentially unreliable measurements, such as low mass, high mass and silent peak overlap of CpG units. The CpG units that failed to produce data for more than 30% of samples (unreliable CpG units) and samples lacking more than 30% of their data points (unreliable samples) were excluded [12].

**Table 1:** Details of measured amplicons and PCR primers.

Gene	Forward primer^1^	Reverse primer^2^	Product length (bp)	CpG unit	CpG site
LINE-1	TTTTATTAGGGAGTGTTAGATAGTGGG	CAAAAACAAACAAACCTCCTTAAAC	430	23	28
MTHFR	GTTTGTAGTTATTTTTGGTTTTAGTTTT	TAACCTAAATTCTCCCTCAAATTCC	443	20	24

*Abbreviations*: LINE-1: long interspersed nucleotide element-1; MTHFR: methylenetetrahydrofolate reductase.

^1^10mer space tag is added at the 5’ primer end with the following sequence: 5’- aggaagagag+primer

^2^T7 promoter is added at the 5’ primer end with the following sequence: 5’- cagtaatacgactcactatagggagaaggct+primer

### Statistical analysis

Analyses of parametric variables were performed using one-way analysis of variance (ANOVA) with a post hoc Bonferroni’s test. In analyses of non-parametric variables, a Kruskal-Wallis test with post hoc Mann-Whitney U-test was employed. Spearman’s rank correlation was used to examine the relationship between the two continuous variables. A p-value < 0.05 was considered to be statistically significant. All statistical tests were conducted using SPSS version 17.0. Post-hoc power analysis was performed by using SAS 9.1.

## Results

### Demographic features of the subjects

**Table 2** presents the baseline characteristics of the 90 subjects included in the present study. Thirty patients with epilepsy (15 males and 15 females with an average±S.D age of 27.2±8.4 years) who had been treated with LTG monotherapy and 30 patients with epilepsy (15 males and 15 females with an average±S.D age of 24.4±8.9 years) who had been treated with VPA monotherapy, and 30 healthy controls (15 males and 15 females with an average±S.D age of 26.1±3.9 years) were enrolled. Age and gender differences were similar between the study groups. The duration of AED therapy at the time of study in the LTG group was 17(6,30) months and 22.5(12.25,59.5) months for the VPA group. The dosage of monotherapy in the LTG group was 50–225mg/day; VPA, 500–1500mg/day. The AED serum concentration in the LTG group was 2.89±1.33μg/ml; VPA, 64.22±29.52μg/ml.

**Table 2:** Demographic features of all the subjects.

	LTG (n = 30)	VPA (n = 30)	Control (n = 30)
Age (year)^a^	27.2±8.4	24.4±8.9	26.1±3.9
Gender (female;male)	15;15	15;15	15;15
Duration of AED therapy (month)^b^	17(6,30)	22.5(12.25,59.5)	
Dosage of AED (mg/day)^b^	112.5(100,181.25)	750(625,1000)	
AED concentration (μg/ml)^a^	2.89±1.33	64.22±29.52	

*Abbreviations*: LTG: lamotrigine; VPA: valproate; AED: antiepileptic drug.

^a^Values expressed as mean ± standard deviation (SD) or

^b^median (interquartile range, IQR)

### OCM nutrient levels in patients with epilepsy and controls

**Fig 1:**
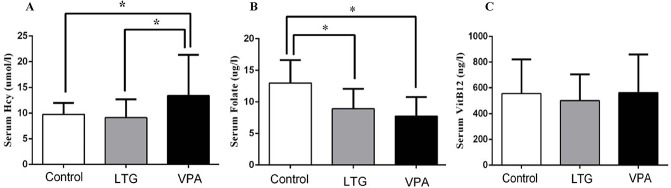
Comparison of serum OCM nutrient levels among the control, LTG and VPA groups. A serum Hcy levels, B serum FA levels and C serum VitB12 levels. *P<0.05. *Abbreviations*: Hcy: homocysteine; FA: folate; VitB12: vitamin B12; LTG: lamotrigine; VPA: valproate.

Concerning the VPA group, serum Hcy levels were significantly higher than that of controls (13.44±7.90 μmol/l vs 9.78±2.22 μmol/l, p = 0.022) (Fig 1A), and serum FA levels were significantly lower than that of the control group (7.75±2.30 μg/l vs 12.98±3.66 μg/l, p<0.001) (Fig 1B), while serum VitB12 levels were indifferent from that of controls (562.50±296.76 ng/l vs 555.03±266.33 ng/l, p>0.05) (Fig 1C).

Compared to the control group, serum Hcy and VitB12 were not different in LTG group (Hcy: 9.13±3.56 μmol/l vs 9.78±2.22 μmol/l, VitB12: 500.00±205.83 ng/l vs 555.03±266.33 ng/l, p>0.05) (Fig 1A and 1C), whereas serum FA levels in the LTG group were significantly lower than in the control group (8.90±3.17 μg/l vs 12.98±3.66 μg/l, p<0.001) (Fig 1B).

### Methylation status of LINE-1 in patients with epilepsy and controls

**Fig 2:**
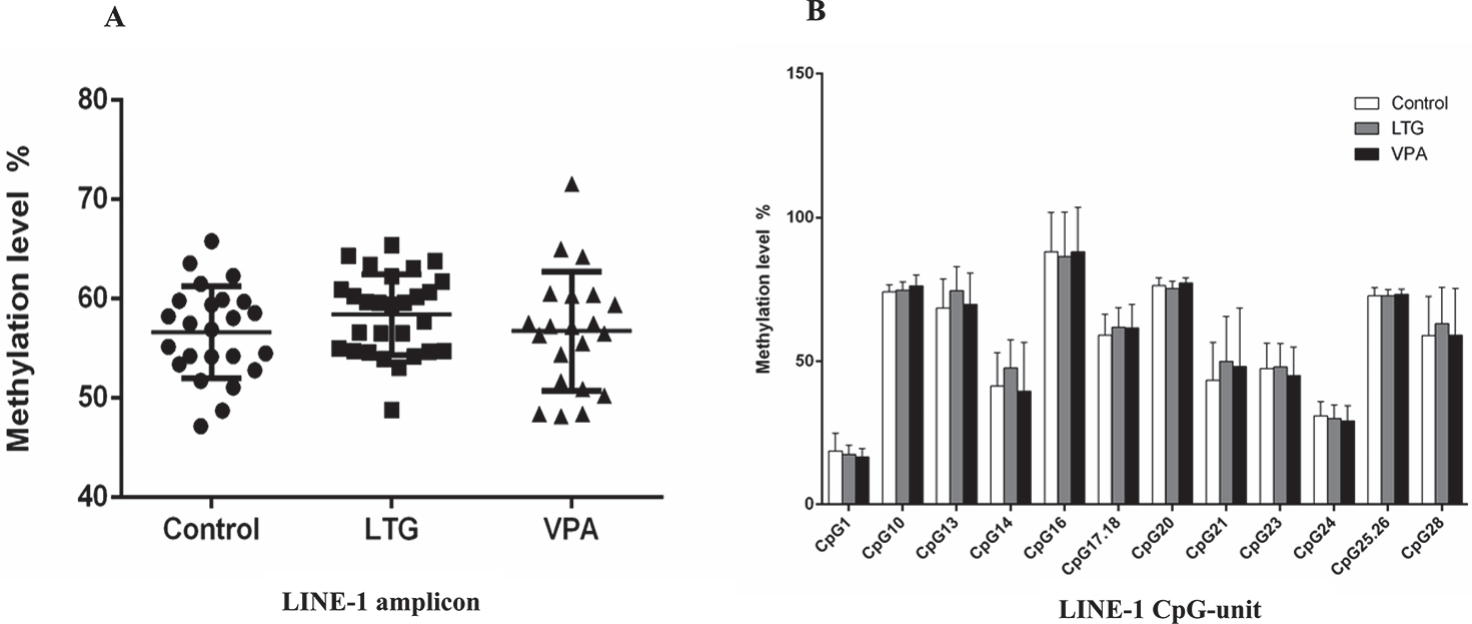
A Methylation levels of LINE-1 among the control, LTG and VPA groups. B Methylation levels of informative CpG units in LINE-1 among the control, LTG and VPA groups. *P<0.05. *Abbreviations*: *LINE-1*: long interspersed nucleotide element-1; LTG: lamotrigine; VPA: valproate.

To determine the whole-genome methylation level, we analyzed the methylation status of the LINE-1 elements in patients with epilepsy and control subjects. The amplicon detected in the 5’-UTR of LINE-1 was 430 base pairs in length and contained 28 CpG sites that could be divided into 23 CpG units. We compared the level of LINE-1 methylation in patients with epilepsy who received AED monotherapy (VPA group and LTG group) with the levels in controls, and did not observed any significant differences (56.75%±5.98% vs 56.62%±4.62%, 58.41%±4.07 vs 56.62%±4.62%, respectively; p>0.05) (Fig 2A).

The methylation status of each CpG unit was also evaluated. After the removal of unreliable data, we obtained 12 informative CpG units containing 14 CpG sites in LINE-1. No significant differences were found in all CpG units between patients who received AED monotherapy and controls (Fig 2B).

### Methylation status of MTHFR in patients with epilepsy and controls

**Fig 3:**
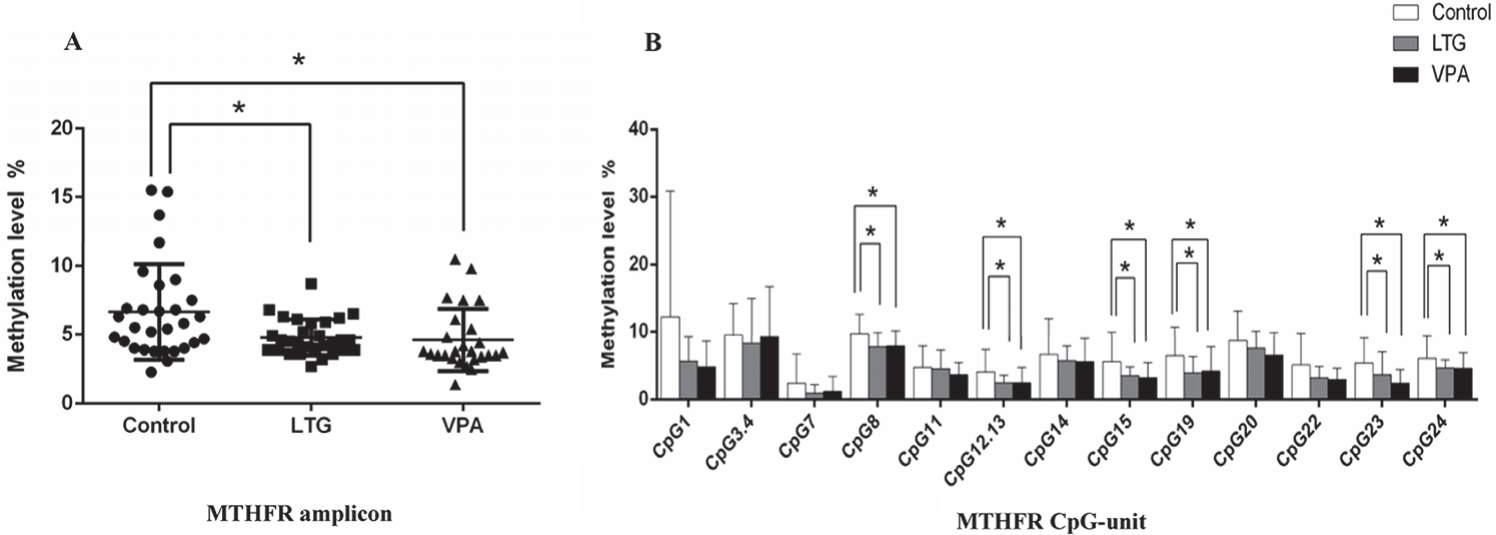
A Methylation levels of the MTHFR amplicon among the control, LTG and VPA groups. B Methylation levels of informative CpG units in the MTHFR amplicon among the control, LTG and VPA groups. *P<0.05. *Abbreviations*:*MTHFR*: methylenetetrahydrofolate reductase; LTG: lamotrigine; VPA: valproate.

The methylation level of the MTHFR amplicon was significantly lower in LTG and VPA groups relative to those in the controls (4.79%±1.30% vs 6.66%±3.47%, p = 0.006; 4.62%±2.24% vs 6.66%±3.47%, p = 0.003, respectively) (Fig 3A).

The methylation level of each CpG unit was also evaluated. After the removal of unreliable data, we obtained 13 informative CpG units containing 15 CpG sites in MTHFR. The methylation levels at CpG_8, CpG_12.13, CpG_15, CpG_19, CpG_23 and CpG_24 were significantly lower in patients with epilepsy who received LTG or VPA monotherapy than those in the controls (p<0.05). No significant differences were found in other CpG units (p>0.05) (Fig 3B).

### Correlation between methylation status and OCM nutrients levels

To investigate the relationship between OCM nutrient levels and the methylation status of the MTHFR amplicon, a correlation analysis was performed. There was a positive correlation between serum FA levels and MTHFR amplicon methylation status (r = 0.25, p = 0.023). No correlation was found between MTHFR methylation and other OCM nutrients, with the exception of FA (r = -0.157, p = 0.15; r = 0.04,p = 0.707, respectively).

### Post-hoc power analysis

Post-hoc power analysis was performed for all tested variables in order to assess the likelihood that we missed detecting a clinically significant difference. Power analysis demonstrated a statistical power value greater than 99% for FA, Hcy and MTHFR methylation, but the power values of VitB12 (power = 12.8%) and LINE-1 methylation (power = 29.3%) was less than 80%.

## Discussion

The present research confirms previously published data that showed that patients with epilepsy undergoing chronic VPA therapy were more prone to develop hyperhomocysteinemia and low folate levels relative to the general population [5, 16]. A novel finding derived from the present study was that hypomethylation levels of the MTHFR amplicon were present in patients with epilepsy who received VPA or LTG monotherapy. To our knowledge, these findings represented the first published study in which methylation levels of LINE-1 and the MTHFR amplicon were compared between AED-treated patients with epilepsy and controls.

Hcy, a thiol-containing amino acid formed by demethylation of methionine, is an intermediate product in OCM. FA and VitB12 are cofactors of OCM. The increased Hcy concentration may be due to a deficiency in cofactors necessary for OCM [17]. The present study demonstrated that patients with epilepsy taking VPA have increased plasma levels of homocysteine. To better understand the cause of the increase in Hcy level, the levels of serum FA and VitB12 were also determined. We found significantly lower concentrations of serum FA in VPA-treated patients with epilepsy compared to that of the controls. In contrast to inducer AEDs that have various effects on enzyme induction in the liver with FA, VPA has less enzyme-inducing activity, but it can impair the intestinal absorption of dietary FA [18]. Moreover, an unexpected but intriguing finding was that treatment with LTG can also result in a significant decrease in FA with unchanged Hcy. Yoo and Hong found that the homozygous thermolabile genotype of MTHFR was an independent predictor of hyperhomocysteinemia in AED-treated patients with epilepsy, suggesting a gene-drug interaction as a cause of hyperhomocysteinemia [19]. The different distribution of MTHFR genotypes may be the cause of different serum Hcy levels between the VPA and LTG groups. Our findings are not consistent with those of previous studies, which showed that most patients under monotherapy with LTG had normal FA levels [6, 7]. As a dihydrofolate reductase inhibitor, LTG decreases fetal FA levels in rats [20].Whether LTG interferes with FA metabolism in humans needs further study with larger samples.

As an intermediate product of OCM, Hcy is recycled back to methionine in the presence of OCM cofactors such as FA and VitB12. Successful cycling of methionine from Hcy ensures provision of the universal methyl-donor S-adenosylmethionine (SAM) for subsequent methylation reactions, including DNA methylation [21]. In contrast with genetic sequence variants, DNA methylation is dynamic and is influenced by many factors, including lifestyle and environment. To assess the stability of DNA methylation across the genome over time, Shah et al. estimated methylation repeatability in peripheral blood and found that a large number of CpGs across the genome, as a result of environmental constraints, have stable DNA methylation variation over the human lifetime [22]. Recently, Smith et al. showed that DNA methylation decreased in neonates of mothers who took AEDs during pregnancy [14]. In the present study, we found that the methylation level of LINE-1 showed no significant difference between AED monotherapeutic patients with epilepsy and controls. Our findings were in accordance with a study conducted by Emes et al., which indicated that the levels of LINE-1 methylation in cord blood were not significantly different between the AED and control groups [23]. We also found that the levels of methylation of MTHFR amplicon in AED-treated patients with epilepsy were significantly lower than that of controls. Lower DNA methylation levels could indicate higher activity of the gene. The increased MTHFR enzymatic activity after VPA treatment has been demonstrated both in vitro and in vivo [24]. A reduction in FA concentration or in its conversion to one-carbon methyl donors can alter the methylation of specific regions of DNA [25]. In the present study, the FA level was significantly lower in the VPA and LTG groups relative to controls, and a positive correlation between FA concentrations and MTHFR methylation levels was observed. As a key enzyme in FA metabolism, MTHFR affects the distribution of FA forms. We hypothesize that higher activity of MTHFR, as observed upon treatment with VPA or LTG, alters the form of FA required for OCM when FA is not sufficient.

An interesting finding of the present study was that serum Hcy levels were significantly higher in the VPA group but not in the LTG group, compared to that of controls. Elevated circulating Hcy is a well-established risk factor for vascular disease such as stroke, myocardial infarction and peripheral arterial disease [26]. Recently, Chuang et al. found that long-term monotherapy with VPA is associated with hyperhomocysteinemia, which leads to an increase in the risk of atherosclerosis in patients with epilepsy, which was not shown for the LTG monotherapy group [27]. Maternal hyperhomocysteinemia as a sensitive biomarker of a dysfunctional OCM has been associated with an increased risk of NTDs in offspring [28]. One of the proposed underlying mechanisms of this association is the induction of alterations in DNA methylation of genes implicated in neural tube development. Stolk et al. conducted a case-control study and found an association between relatively low MTHFR amplicon methylation levels and NTDs[29]. In present study, higher homocysteine levels and lower MTHFR amplicon methylation levels were found in VPA monotherapy group. Popular epilepsy and pregnancy registries have stated that first trimester exposure to VPA increases the risk of NTDs from approximately 1 per 1,000 to 10 per 1,000 births [30, 31]. The mechanisms by which VPA exerts the teratogenic effect remain unclear. Further research is required to identify whether VPA affects embryonic and neural tube development by effecting DNA methylation.

Limitations of this research should be considered. First, the small number of patients recruited to each AED group was insufficient to draw a firm conclusion. Post-hoc power analysis indicated that our research had sufficient to moderate power to detect significant group differences for FA, Hcy concentrations and MTHFR methylation respectively, but that larger sample sizes were warranted to detect significant group differences in VitB12 concentration and LINE-1 methylation. Second, a possible interaction between the epilepsy syndromic category and one-carbon metabolic changes caused by each AED was not contemplated in our study. Third, the sensitivity of the method we used to detect DNA methylation levels was insufficient. It is known that the MassARRAY technique has a sensitivity level of approximately 5%, while some of our results of MTHFR methylation were below this level. Finally, because we conducted a cross-sectional study, we could not exclude other environmental factors that might affect the results.

In conclusion, our findings suggest that AED monotherapy may induce specific regions of DNA hypomethylation through their effects on OCM. Patients undergoing chronic AED treatment should be routinely screened for FA and VitB12 deficiencies and hyperhomocysteinemia, and any vitamin deficiency should be corrected when necessary.
